# Supplementary material for: Optimisation of Glycerol and Itaconic Anhydride Polycondensation
Source: Molecules. 2022 Jul 20;27(14):4627. doi: 10.3390/molecules27144627 (PMC9324205; doi:10.3390/molecules27144627)
Supplement: Supplementary file 1 [file molecules-27-04627-s001.zip › molecules-1814508-supplementary.pdf]

Supplementary information

Optimisation of glycerol and itaconic anhydride polycondensation

Krzysztof Kolankowski, Magdalena Miętus, Paweł Ruśkowski, Agnieszka Gadomska-Gajadhur

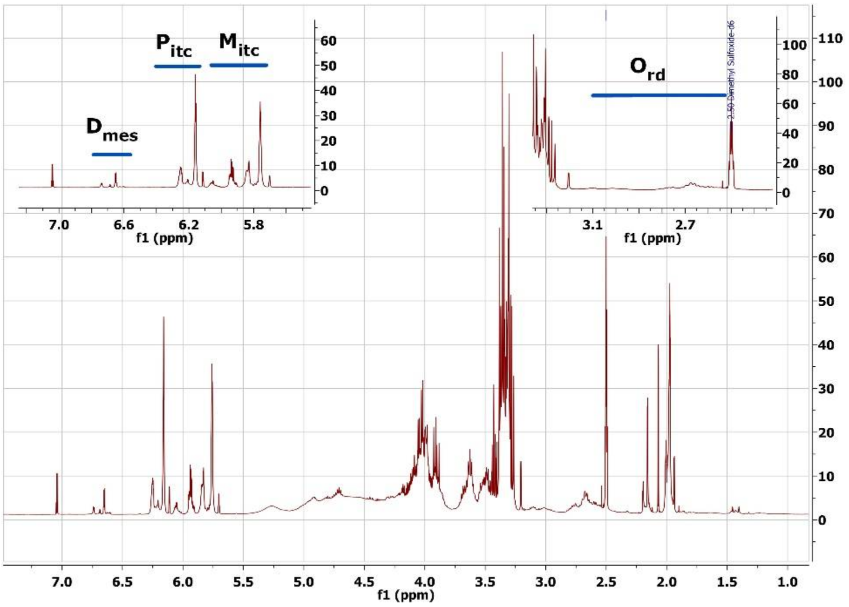

Figure S1. The ^{1}H NMR spectra of poly(glycerol itaconate) – side reaction calculations

$$\text{Ordelt saturation} = (\int O_{rd}/3) / ((\int O_{rd}/3)+(\int M_{itc}/2 + \int P_{itc}/2+\int D_{Mes})) * 100\%$$

$\int O_{rd}$ - the value of the integral of the signal from the Ordelt's protons

$\int M_{itc}$ - the value of the integral of the signal from the itaconic monoesters

$\int P_{itc}$ - the value of the integral of the signal from the itaconic polyesters, oligoesters, monoesters

$\int D_{Mes}$ - the value of the integral of the signal from the mesaconic derivatives

$$\text{Isomerisation to mesaconic fragments} = (\int D_{Mes}) / ((\int D_{Mes})+(\int M_{itc}/2 + \int P_{itc}/2)) * 100\%$$

$\int D_{Mes}$ - the value of the integral of the signal from the mesaconic derivatives

$\int M_{itc}$ - the value of the integral of the signal from the itaconic monoesters

$\int P_{itc}$ - the value of the integral of the signal from the itaconic polyesters, oligoesters, monoesters

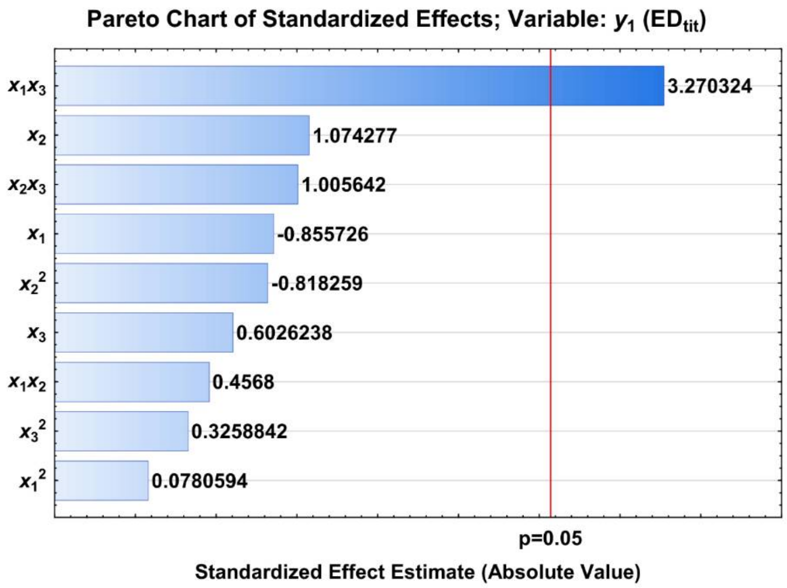

Figure S2. Pareto chart for the ED_{titr} variable

$$y_1 = 49{,}4 + 14{,}4 x_1 x_3$$

Table S1. Significance test of regression equation coefficients for the ED_{titr} variable

Input variables/ Parameter relation	Regression coefficient	Standard error	t(5)	p	-95,00% Confidence limit	95,00% Confidence limit
Constant	0,4937	0,01922	25,68	0,000000	0,4522	0,5352
x_1 to x_3	0,1439	0,03722	3,270	0,001949	0,06348	0,2243

Table S2. Model adequacy test for the ED_{titr} variable

Input variables/ Parameter relation	SS	df	MS	F	p
x_1 to x_3	0,08282	1	0,08283	14,94	0,001949
Standard error	0,07206	13	0,005543		
Total SS	0,1549	14			

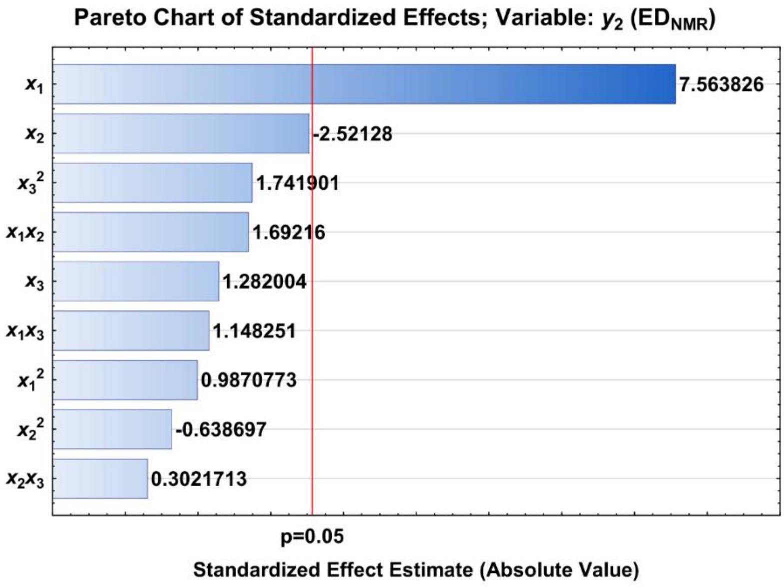

Figure S3. Pareto chart for the ED_{NMR} variable

$$y_2 = 68{,}2 + 2{,}21x_1 - 0{,}738x_2$$

Table S3. Significance test of regression equation coefficients for the ED_{NMR} variable

Input variables/ Parameter relation	Regression coefficient	Standard error	t(5)	p	-95,00% Confidence limit	95,00% Confidence limit
Constant	0,6820	0,004777	143,8	0,000000	0,6747	0,6993
x_1	0,02213	0,002925	7,564	0,0006400	0,01461	0,029644
x_2	-0,007375	0,002925	-2,521	0,05309	-0,01489	0,0001440

Table S4. Model adequacy test for the ED_{NMR} variable

Input variables/ Parameter relation	SS	df	MS	F	p
x_1	0,003916	1	0,003916	44,62	0,0000230
x_2	0,000435	1	0,0004350	4,958	0,04588
Standard error	0,001053	12	0,00008800		
Total SS	0,005404	14			

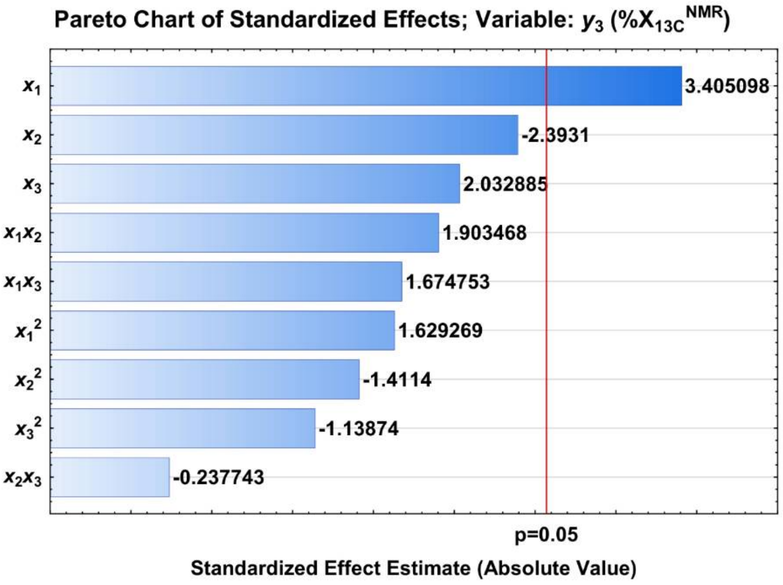

Figure S4. Pareto chart for the $\%X_{13C}^{NMR}$ variable

$$y_3 = 57{,}1 + 5{,}35x_1 - 3{,}76x_2$$

Table S5. Significance test of regression equation coefficients for the $\%X_{13C}^{NMR}$ variable

Input variables/ Parameter relation	Regression coefficient	Standard error	t(5)	p	-95,00% Confidence limit	95,00% Confidence limit
Constant	0,5722	0,02566	21,86	0,000004	0,4949	0,6268
x_1	0,05350	0,01571	3,405	0,01914	0,01311	0,09389
x_2	-0,03760	0,01571	-2,393	0,06215	-0,07798	0,002788

Table S6. Model adequacy test for the $\%X_{13C}^{NMR}$ variable

Input variables/ Parameter relation	SS	df	MS	F	p
x_1	0,02290	1	0,02290	6,326	0,02715
x_2	0,01131	1	0,01131	3,125	0,1025
Standard error	0,04343	12	0,003619		
Total SS	0,07763	14			

Table S7. Values of output variables to generate the response utility profile

Utility	Output variable		
	ED_{tit} [%]	ED_{NMR} [%]	$\%X_{13C}^{NMR}$
Low	67,8	70,7	74,0
Medium	83,9	85,4	87,0
High	100,0	100,0	100,0

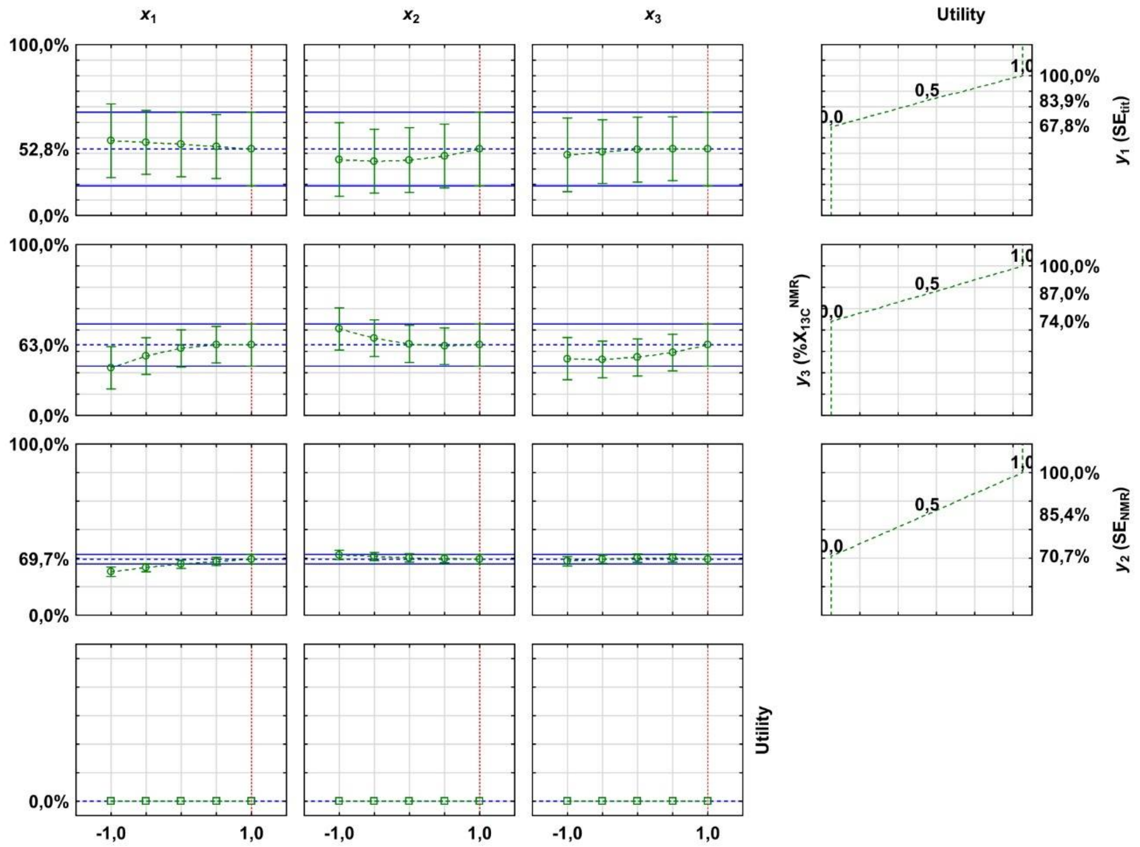

Figure S5. Profile of approximated values of input variables and utility
